# Development and validation of a survival prediction model for patients with advanced non-small cell lung cancer based on LASSO regression

**DOI:** 10.3389/fimmu.2024.1431150

**Published:** 2024-08-02

**Authors:** Yimeng Guo, Lihua Li, Keao Zheng, Juan Du, Jingxu Nie, Zanhong Wang, Zhiying Hao

**Affiliations:** ^1^ Department of Pharmacy, Shanxi Province Cancer Hospital/Shanxi Hospital Affiliated to Cancer Hospital, Chinese Academy of Medical Sciences/Cancer Hospital Affiliated to Shanxi Medical University, Taiyuan, Shanxi, China; ^2^ School of Pharmacy, Shanxi Medical University, Taiyuan, China; ^3^ Department of Obstetrics and Gynecology, Shanxi Bethune Hospital/Shanxi Academy of Medical Sciences/Tongji Shanxi Hospital/Third Hospital of Shanxi Medical University, Taiyuan, Shanxi, China

**Keywords:** non-small cell lung cancer, LASSO regression, nomogram, prediction model, random survival forest

## Abstract

**Introduction:** Lung cancer remains a significant global health burden, with non-small cell lung cancer (NSCLC) being the predominant subtype. Despite advancements in treatment, the prognosis for patients with advanced NSCLC remains unsatisfactory, underscoring the imperative for precise prognostic assessment models. This study aimed to develop and validate a survival prediction model specifically tailored for patients diagnosed with NSCLC. Methods: A total of 523 patients were randomly divided into a training dataset (n=313) and a validation dataset (n=210). We conducted initial variable selection using three analytical methods: univariate Cox regression, LASSO regression, and random survival forest (RSF) analysis. Multivariate Cox regression was then performed on the variables selected by each method to construct the final predictive models. The optimal model was selected based on the highest bootstrap C-index observed in the validation dataset. Additionally, the predictive performance of the model was evaluated using time-dependent receiver operating characteristic (Time-ROC) curves, calibration plots, and decision curve analysis (DCA). Results: The LASSO regression model, which included N stage, neutrophil-lymphocyte ratio (NLR), D-dimer, neuron-specific enolase (NSE), squamous cell carcinoma antigen (SCC), driver alterations, and first-line treatment, achieved a bootstrap C-index of 0.668 (95% CI: 0.626-0.722) in the validation dataset, the highest among the three models tested. The model demonstrated good discrimination in the validation dataset, with area under the ROC curve (AUC) values of 0.707 (95% CI: 0.633-0.781) for 1-year survival, 0.691 (95% CI: 0.616-0.765) for 2-year survival, and 0.696 (95% CI: 0.611-0.781) for 3-year survival predictions, respectively. Calibration plots indicated good agreement between predicted and observed survival probabilities. Decision curve analysis demonstrated that the model provides clinical benefit at a range of decision thresholds. Conclusion: The LASSO regression model exhibited robust performance in the validation dataset, predicting survival outcomes for patients with advanced NSCLC effectively. This model can assist clinicians in making more informed treatment decisions and provide a valuable tool for patient risk stratification and personalized management.

## 1 Introduction

According to the latest 2024 International Agency for Research on Cancer (IARC) cancer burden report, an estimated 2,480,100 people globally were expected to be diagnosed with lung cancer in 2022, making up one-eighth of all new cancer cases. Furthermore, lung cancer was anticipated to be the leading cause of cancer-related deaths, with an estimated 1,817,500 fatalities (1). Despite advances in detection and treatment, the subtle early symptoms and high metastatic potential of lung cancer mean many cases are still diagnosed at an advanced stage.

In the field of lung cancer, non-small cell lung cancer (NSCLC) is the predominant subtype, accounting for approximately 85% of all cases (2). Despite continuous advancements in medical technology that have contributed to prolonging survival time among patients with advanced NSCLC, overall prognosis remains unsatisfactory. Therefore, precise prognostic assessment assumes paramount importance for physicians in devising targeted treatment strategies. Moreover, it plays a vital role in predicting patients’ quality of life, survival time, and evaluating their eligibility for participation in clinical trials.

In recent years, a multitude of clinical prediction models have been developed to evaluate the prognosis of patients with various tumor types, including colorectal cancer (3), ovarian cancer (4), and liver cancer (5), among others. In the field of lung cancer research, scholars have also constructed prognostic models for advanced NSCLC. Hoang and colleagues conducted an analysis of data from two phase III randomized clinical trials, where they identified metastasis status, performance status scores, appetite, and surgical history as significant prognostic factors for patients with non-small cell lung cancer undergoing first-line platinum-based doublet chemotherapy. Subsequently, they developed a prognostic model to assess the 1-year and 2-year survival rates of these patients (6). Furthermore, Tao Wang and his team utilized data from three randomized controlled trials to construct a prognostic model incorporating nine variables: sex, histological type, ECOG performance score, peritoneal metastasis, skin metastasis, liver metastasis, hemoglobin levels, white blood cell count, lymphocyte percentage. This model has demonstrated efficacy in predicting the survival of patients with advanced lung cancer over a period ranging from 6 to 18 months (7). These models serve as valuable references for devising treatment strategies for patients with advanced lung cancer. However, in practical applications, these prognostic models may encounter several limitations. For instance, the exclusion of potentially valuable clinical data such as genetic information and specific laboratory test results can significantly impact the accuracy of the study. Additionally, the model data primarily originates from specific randomized controlled clinical trials with stringent inclusion and exclusion criteria, which might not fully capture the characteristics of the entire NSCLC patient population. Moreover, the emergence of novel treatment methods can significantly impact patient prognosis. Given these constraints, there is an urgent need to develop new prognostic models that comprehensively integrate clinical, pathological, molecular biological, and treatment parameters while employing advanced statistical techniques to achieve a more precise assessment of the prognosis in patients with advanced lung cancer.

With the advancement of bioinformatics and statistical methodologies, a range of sophisticated statistical techniques, such as Cox regression, least absolute shrinkage and selection operation (LASSO) regression, and random survival forest (RSF), have been employed in constructing prognostic models (8–10). These approaches aim to analyze and integrate extensive clinical data for identifying crucial factors influencing the prognosis of patients with advanced NSCLC, thereby facilitating more precise treatment recommendations. This study aims to compare the efficacy of these methods in prognostic assessment for advanced NSCLC to determine the optimal prognostic model, ultimately providing a more scientifically grounded basis for clinical decision-making and enhancing treatment outcomes and quality of life among patients with advanced NSCLC.

## 2 Materials and methods

### 2.1 Patients and clinicopathological data collection

This retrospective study was conducted at Shanxi Province Cancer Hospital using data from their Electronic Medical Record system. Survival data were obtained from the hospital’s affiliated follow-up center. The data collection was between January 2019 and December 2020. The study included patients who met the following criteria (1) Patients with histologically confirmed advanced NSCLC, classified according to the 8th edition of the American Joint Committee on Cancer (AJCC) staging system as stage IV, who are undergoing initial treatment; (2) age 18 years or older; (3) ECOG score of 0-2; (4) receipt of at least 4 cycles of systemic therapy; and (5) availability of complete baseline clinical and laboratory data. The exclusion criteria were: (1) age less than 18 years; (2) disease stage I-III; (3) previous or relevant history of other malignancies; (4) withdrawal from treatment after diagnosis; (5) receipt of fewer than 4 cycles of systemic therapy; and (6) incomplete clinical data or loss to follow-up. To ensure model reliability and predictive accuracy, a minimum of 10 events per predictor variable (10EPV) is recommended (11). This principle aims to decrease the likelihood of overfitting and enhance the model’s capacity to generalise to independent datasets. The eligible patients were randomly divided into training and validation datasets in a 6:4 ratio. The training dataset was used for constructing the model, while the validation dataset was used for validating. The study’s main outcome was overall survival (OS), which is defined as the time from the date of the tumour’s pathological diagnosis to the patient’s death or the end of follow-up, whichever occurred first. Follow-up ended on 31 December 2023. This study received ethical approval from the Shanxi Province Cancer Hospital Ethical Review Board (No. KY2024053). Due to the retrospective design of the study, the requirement for informed consent was waived by the ethics committee.

The study extracted baseline clinicopathological characteristics from patients who met the inclusion and exclusion criteria. Laboratory tests included lactate dehydrogenase, serum albumin, neutrophils, monocytes, lymphocytes, platelets, D-dimer, and fibrinogen. Clinicopathological data were collected including age, sex, height, weight, smoking status, comorbidities, tuberculosis history, family history, histological type of tumor, presence of pleural effusion, tumor markers, distant metastatic status, gene mutation status, and first-line treatment. Five composite indices were constructed based on laboratory examination parameters, namely: neutrophil-lymphocyte ratio (NLR), monocyte-lymphocyte ratio (MLR), platelet-lymphocyte ratio (PLR), prognostic-nutritional index (PNI), and albumin-fibronectin ratio (AFR) (12–15).

### 2.2 Statistical analysis

Continuous variables, including D-dimer, NLR, MLR, PLR, PNI, AFR, lactate dehydrogenase (LDH), carcinoembryonic antigen (CEA), neuron-specific enolase (NSA), squamous cell carcinoma antigen (SCC), carbohydrate antigen 125 (CA125), and carbohydrate antigen 19-9 (CA19-9), were dichotomized at inflection points determined by receiver operating characteristic (ROC) curve analysis. Continuous variables were presented as either mean ± standard deviation or median with interquartile range. Comparisons between groups were conducted using either Student’s t-test or the Wilcoxon rank-sum test, depending on the data distribution. Categorical variables were reported as counts and percentages, with group comparisons performed using the chi-square test.

In this study, we conducted initial variable selection using three analytical methods: univariate Cox regression, LASSO regression, and RSF. To avoid prematurely excluding potentially important variables, those with a p-value less than 0.1 in the univariate Cox regression analyses were selected for inclusion in subsequent multivariable analyses. Shapley additive explanations (SHAP), which draw upon the classical Shapley values from game theory (16), are widely employed for interpreting complex machine learning models. In this study, we utilized SurvSHAP(t), an extension of SHAP specifically designed for survival models (17), to interpret the impact of predictor variables selected by the RSF on the survival function. Initially, we used RSF to screen and select predictor variables most relevant to survival outcomes. Subsequently, we employed SHAP values to quantify the contribution of each selected variable to the model’s predictions. This allowed us to rank the variables by importance, providing clear insights into which factors most substantially impact survival predictions. Combining RSF and SHAP methodologies offered a robust framework for variable selection and interpretation. RSF isolated the most predictive variables, while SurvSHAP(t) quantified their contributions, resulting in an importance ranking that elucidates each predictor’s role in survival analysis.

The variables initially selected by these three methods were then individually subjected to multivariable Cox regression analysis to determine the final set of variables to be included. Based on the selected variables, we subsequently developed Cox regression, LASSO regression, and RSF models, respectively.

To evaluate the predictive accuracy of the models in survival analysis, the concordance index (C-index) was calculated using 500 bootstrap samplings. This approach not only evaluates the models’ predictive accuracy but also provides insights into their stability and generalizability across different sample sets. The optimal model was selected based on the highest bootstrap C-index observed in the validation dataset. To further assess the model’s performance, we generated time-dependent receiver operating characteristic (Time-ROC) curves, calibration curves, and decision curves analysis(DCA). After developing the model, we calculated the risk score for each patient by inputting their respective variables into the model. We then determined the median risk score for the entire patient cohort. Patients were subsequently classified into high-risk and low-risk groups based on whether their individual risk score was above or below the median risk score. To estimate survival rates for the high-risk and low-risk groups, we employed the Kaplan-Meier method. Additionally, we evaluated the differences in survival curves between the two groups using the log-rank test. This approach allowed us to assess the prognostic value of the risk scores effectively.

The statistical tests conducted in this study were two-tailed, and a significance level of P<0.05 was adopted to determine statistical significance. Statistical analyses were performed using R version 4.2.1, employing specific packages for different models: “survival” for Cox regression, “glmnet” for LASSO regression, “randomForestSRC” for RSF, “survex” for SurvSHAP(t), “survivalROC” for Time-ROC curves, “rms” for nomograms, “pec” for calibration and Time-AUC curves, “dcurves” for clinical decision curves, and “survivalminer” for risk-stratified KM curves.

## 3 Results

### 3.1 Characteristics of study patients

**Figure 1:**
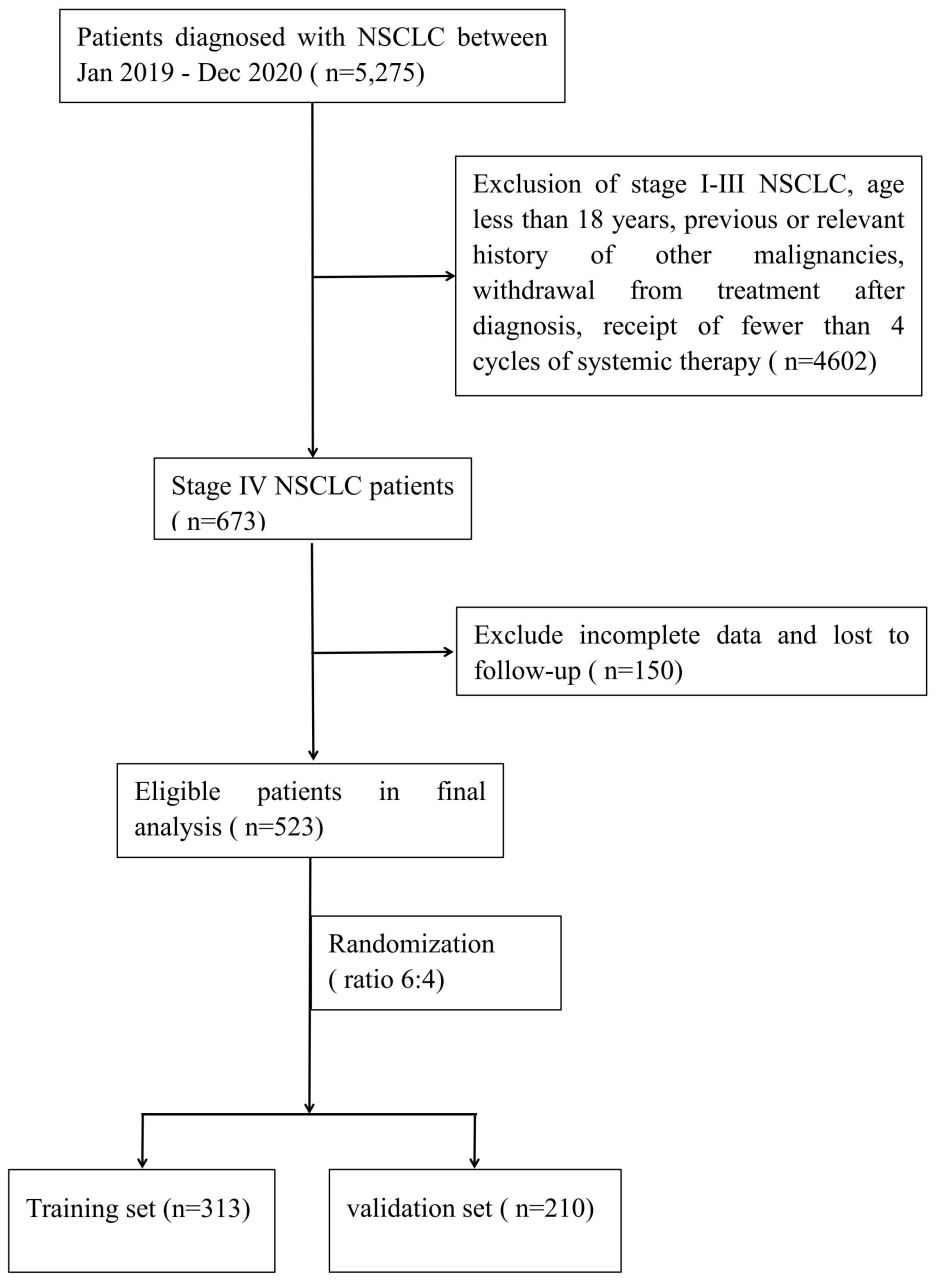
Flowchart for retrospective study selection.

A total of 523 patients with advanced NSCLC were included in this study. These patients were then randomly divided into two datasets at a ratio of 6:4, resulting in a training dataset consisting of 313 patients and a validation dataset comprising 210 patients (see **Figure 1**). Among the patients included in the study, 64.44% were male and 35.56% were female. Patients aged 60 years or older constituted 54.68% of the sample, and 53.35% had a history of smoking. The proportions of patients with liver and brain metastases were 13.58% and 27.72%, respectively. Actionable oncogenic driver alterations involving genes such as *EGFR, ALK, MET, KRAS, BRAF*, and *ROS1* were observed in 54.49% of the cases. Regarding first-line treatment strategies, 44.36% of patients received chemotherapy, 43.40% underwent targeted therapy, including single-agent tyrosine kinase inhibitors (TKIs) or a combination of TKIs and chemotherapy. Immunotherapy, either as monotherapy or in combination with chemotherapy, was administered to 12.24% of patients. The median follow-up period for this study was 23 months, at the end of which 78.20% of patients had died. A comparison between the training and validation datasets showed no statistically significant differences in clinicopathological characteristics, ensuring comparability of the datasets for subsequent analyses. Detailed statistical analysis results are presented in **Table 1**.

**Table 1:** Clinical characteristics of patients with advanced NSCLC.

Characteristics	[ALL] N=523	validation set N=210	Training set N=313	P value
Sex, n (%)				0.684
Female	186 (35.56%)	72 (34.29%)	114 (36.42%)	
Male	337 (64.44%)	138 (65.71%)	199 (63.58%)	
Age, (years), n (%)				0.766
<60	237 (45.32%)	93 (44.29%)	144 (46.01%)	
≥60	286 (54.68%)	117 (55.71%)	169 (53.99%)	
BMI, n (%)				0.796
<18.5	25 (4.78%)	10 (4.76%)	15 (4.79%)	
18.5-24	283 (54.11%)	110 (52.38%)	173 (55.27%)	
>24	215 (41.11%)	90 (42.86%)	125 (39.94%)	
Smoking, n (%)				0.658
No	244 (46.65%)	95 (45.24%)	149 (47.60%)	
Yes	279 (53.35%)	115 (54.76%)	164 (52.40%)	
Respiratory comorbidity, n (%)				0.113
No	474 (90.63%)	196 (93.33%)	278 (88.82%)	
Yes	49 (9.37%)	14 (6.67%)	35 (11.18%)	
Cardiocerebrovascular comorbidity, n (%)				0.416
No	372 (71.13%)	154 (73.33%)	218 (69.65%)	
Yes	151 (28.87%)	56 (26.67%)	95 (30.35%)	
Diabetes comorbidity, n (%)				0.218
No	479 (91.59%)	188 (89.52%)	291 (92.97%)	
Yes	44 (8.41%)	22 (10.48%)	22 (7.03%)	
History of tuberculosis, n (%)				1.000
No	511 (97.71%)	205 (97.62%)	306 (97.76%)	
Yes	12 (2.29%)	5 (2.38%)	7 (2.24%)	
Family history of lung cancer, n (%)				0.081
No	493 (94.26%)	203 (96.67%)	290 (92.65%)	
Yes	30 (5.74%)	7 (3.33%)	23 (7.35%)	
Pleuraleffusion, n (%)				0.825
No	322 (61.57%)	131 (62.38%)	191 (61.02%)	
Yes	201 (38.43%)	79 (37.62%)	122 (38.98%)	
T stage, n (%)				0.820
<T3	246 (47.04%)	97 (46.19%)	149 (47.60%)	
≥T3	277 (52.96%)	113 (53.81%)	164 (52.40%)	
N stage, n (%)				1.000
<N2	110 (21.03%)	44 (20.95%)	66 (21.09%)	
≥N2	413 (78.97%)	166 (79.05%)	247 (78.91%)	
Histological type, n (%)				0.987
Non-squamous	419 (80.11%)	168 (80.00%)	251 (80.19%)	
Squamous	91 (17.40%)	37 (17.62%)	54 (17.25%)	
Unknown	13 (2.49%)	5 (2.38%)	8 (2.56%)	
Number of metastatic organs, n (%)				0.580
<3	421 (80.50%)	172 (81.90%)	249 (79.55%)	
≥3	102 (19.50%)	38 (18.10%)	64 (20.45%)	
Liver metastasis, n (%)				0.118
No	452 (86.42%)	188 (89.52%)	264 (84.35%)	
Yes	71 (13.58%)	22 (10.48%)	49 (15.65%)	
Bone metastasis, n (%)				0.980
No	288 (55.07%)	115 (54.76%)	173 (55.27%)	
Yes	235 (44.93%)	95 (45.24%)	140 (44.73%)	
Brain metastasis, n (%)				0.180
No	378 (72.28%)	159 (75.71%)	219 (69.97%)	
Yes	145 (27.72%)	51 (24.29%)	94 (30.03%)	
NLR, n (%)				0.720
<2.565	213 (40.73%)	88 (41.90%)	125 (39.94%)	
≥2.565	310 (59.27%)	122 (58.10%)	188 (60.06%)	
MLR, n (%)				0.523
<0.415	392 (74.95%)	161 (76.67%)	231 (73.80%)	
≥0.415	131 (25.05%)	49 (23.33%)	82 (26.20%)	
PLR, n (%)				0.294
<172.3	273 (52.20%)	116 (55.24%)	157 (50.16%)	
≥172.3	250 (47.80%)	94 (44.76%)	156 (49.84%)	
AFR, n (%)				0.430
<5.170	15 (2.87%)	8 (3.81%)	7 (2.24%)	
≥5.170	508 (97.13%)	202 (96.19%)	306 (97.76%)	
LDH, (U/L), n (%)				0.583
<230.5	300 (57.36%)	124 (59.05%)	176 (56.23%)	
≥230.5	223 (42.64%)	86 (40.95%)	137 (43.77%)	
PNI, n (%)				0.552
<56.53	472 (90.25%)	192 (91.43%)	280 (89.46%)	
≥56.53	51 (9.75%)	18 (8.57%)	33 (10.54%)	
D-dimer, (ng/ml), n (%)				0.310
<300.0	266 (50.86%)	113 (53.81%)	153 (48.88%)	
≥300.0	257 (49.14%)	97 (46.19%)	160 (51.12%)	
CEA, (μg/L), n (%)				0.577
<0.965	61 (11.66%)	27 (12.86%)	34 (10.86%)	
≥0.965	462 (88.34%)	183 (87.14%)	279 (89.14%)	
NSE, (μg/L), n (%)				0.825
<5.605	322 (61.57%)	131 (62.38%)	191 (61.02%)	
≥5.605	201 (38.43%)	79 (37.62%)	122 (38.98%)	
SCC, (ng/ml), n (%)				0.570
<0.445	399 (76.29%)	157 (74.76%)	242 (77.32%)	
≥0.445	124 (23.71%)	53 (25.24%)	71 (22.68%)	
CA125, (U/ml), n (%)				0.385
<15.79	157 (30.02%)	68 (32.38%)	89 (28.43%)	
≥15.79	366 (69.98%)	142 (67.62%)	224 (71.57%)	
CA19-9, (U/ml), n (%)				0.564
<50.02	408 (78.01%)	167 (79.52%)	241 (77.00%)	
≥50.02	115 (21.99%)	43 (20.48%)	72 (23.00%)	
Ki67, n (%)				0.651
<50%	165 (31.55%)	69 (32.86%)	96 (30.67%)	
≥50%	174 (33.27%)	65 (30.95%)	109 (34.82%)	
Unknown	184 (35.18%)	76 (36.19%)	108 (34.50%)	
driver alterations, n (%)				0.303
Yes	285 (54.49%)	117 (55.71%)	168 (53.67%)	
No	150 (28.68%)	64 (30.48%)	86 (27.48%)	
Unknown	88 (16.83%)	29 (13.81%)	59 (18.85%)	
First-line treatment, n (%)				0.978
Chemotherapy	232 (44.36%)	94 (44.76%)	138 (44.09%)	
Targeted Therapy	227 (43.40%)	91 (43.33%)	136 (43.45%)	
Immunotherapy	64 (12.24%)	25 (11.90%)	39 (12.46%)	
Death, n (%)				0.709
No	114 (21.80%)	48 (22.86%)	66 (21.09%)	
Yes	409 (78.20%)	162 (77.14%)	247 (78.91%)	
time, Median (IR)	23.00 [11.00;38.00]	23.00 [12.00;37.00]	22.00 [11.00;38.00]	0.527

### 3.2 Variable selection and model construction

**Figure 2:**
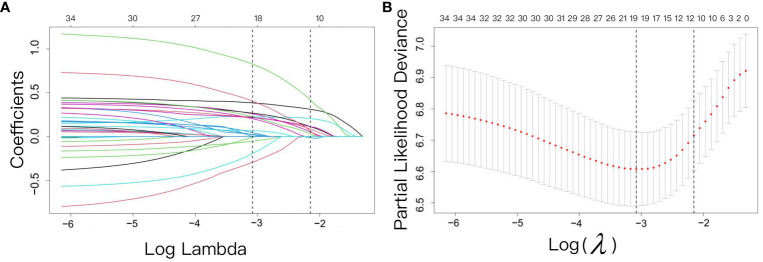
Screening of variables based on LASSO regression. The variation characteristics of the coefficient of variables. **(A)** The variation characteristics of the coefficient of variables; **(B)** The cross-validation method is used to select the optimal value of the parameter λ in the Lasso regression model.

**Figure 3:**
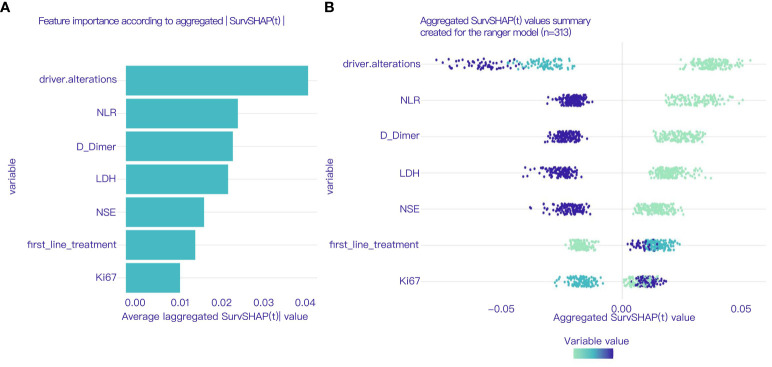
**(A)** Sorts by features importance based on SHAP value. **(B)** Bee swarm plot showing the magnitude and direction of the impact of each variable on the model prediction according to the aggregated SurvSHAP(t) value.

The variable selection process was performed independently using three distinct methodologies: univariate Cox regression, LASSO regression, and RSF analysis, encompassing all variables under investigation. Univariate Cox regression identified 21 variables including BMI, smoking, diabetes comorbidity, history of tuberculosis, T stage, N stage, histological type, liver metastasis, brain metastasis, NLR, MLR, PLR, and LDH, as detailed in **Table 2**. LASSO regression was performed using 10-fold cross-validation, and at a lambda of 1 standard error (λ.1se = 0.117), it selected 10 non-zero coefficients corresponding to 9 variables: N stage, NLR, LDH, D-dimer, NSE, SCC, Ki67, driver alterations, and first-line treatment, as shown in **Figure 2**. After analyzing the results of the RSF using the SurvSHAP(t), we successfully identified seven key variables that significantly influence the prediction outcomes, NLR, D-dimer, LDH, NSE, driver alterations, first-line treatment and N stage. These variables were ranked based on their contribution to the predictive output, highlighting their potential importance in forecasting patient survival rates. Detailed results are presented in **Figure 3**.

**Table 2:** Preliminary variable selection via univariate Cox regression analysis.

characteristics	HR	CI	P
Sex			
Female		Reference	
Male	1.221	0.94-1.585	0.134
Age, (years)			
<60		Reference	
≥60	1.168	0.909-1.5	0.225
BMI			
<18.5		Reference	
18.5-24	0.698	0.378-1.291	0.252
>24	0.757	0.582-0.984	0.038
Smoking			
No		Reference	
Yes	1.446	1.122-1.863	0.004
Respiratory_comorbidity			
No		Reference	
Yes	1.192	0.804-1.768	0.383
Cardiocerebrovascular_comorbidity			
No		Reference	
Yes	1.154	0.88-1.513	0.3
Diabetes_comorbidity			
No		Reference	
Yes	1.496	0.954-2.345	0.079
History of tuberculosis			
No		Reference	
Yes	0.358	0.114-1.118	0.077
Family history of lung cancer			
No		Reference	
Yes	0.722	0.435-1.201	0.21
Pleuraleffusion			
No		Reference	
Yes	0.987	0.764-1.275	0.92
T			
<T3		Reference	
≥T3	1.283	0.999-1.649	0.051
N			
<N2		Reference	
≥N2	1.678	1.206-2.334	0.002
Histological type			
Non-squamous		Reference	
Squamous	1.874	1.37-2.564	0
Unknown	1.433	0.673-3.05	0.35
Number of metastatic organs			
<3		Reference	
≥3	1.374	1.022-1.849	0.035
Liver metastasis			
No		Reference	
Yes	1.457	1.048-2.026	0.025
Bone metastasis			
No		Reference	
Yes	1.129	0.879-1.451	0.342
Brain metastasis			
No		Reference	
Yes	1.291	0.99-1.685	0.06
NLR			
<2.565		Reference	
≥2.565	2.05	1.568-2.682	0
MLR			
<0.415		Reference	
≥0.415	1.64	1.247-2.157	0
PLR			
<172.3		Reference	
≥172.3	1.274	0.992-1.636	0.058
AFR			
<5.170		Reference	
≥5.170	1.534	0.632-3.727	0.344
LDH, (U/L)			
<230.5		Reference	
≥230.5	1.874	1.458-2.408	0
PNI			
<56.53		Reference	
≥56.53	0.699	0.451-1.085	0.11
D-dimer, (ng/ml)			
<300.0		Reference	
≥300.0	1.695	1.316-2.182	0
CEA, (μg/L)			
<0.965		Reference	
≥0.965	1.012	0.669-1.531	0.955
NSE, (μg/L)			
<5.605		Reference	
≥5.605	1.69	1.313-2.176	0
SCC, (ng/ml)			
<0.445		Reference	
≥0.445	1.604	1.201-2.143	0.001
CA125, (U/ml)			
<15.79		Reference	
≥15.79	1.264	0.955-1.672	0.101
CA19-9, (U/ml)			
<50.02		Reference	
≥50.02	1.493	1.12-1.99	0.006
Ki67			
<50%		Reference	
≥50%	1.783	1.3-2.446	0
Unknown	1.105	0.799-1.528	0.546
driver alterations			
Yes		Reference	
No	1.936	1.443-2.598	0
Unknown	2.643	1.909-3.661	0
First-line treatment			
Chemotherapy		Reference	
Targeted Therapy	0.578	0.442-0.755	0
Immunotherapy	0.702	0.464-1.061	0.093

**Figure 4:**
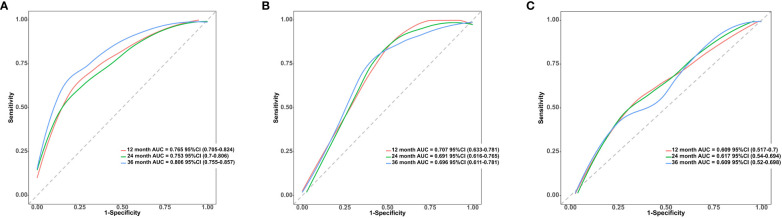
**(A)** ROC curves and AUC for 1-Year, 2-Year, and 3-Year OS predictions using the LASSO regression model in the training cohort. **(B)** ROC curves and AUC for 1-Year, 2-Year, and 3-Year OS predictions using the LASSO regression model in the validation cohort. **(C)** ROC curves and AUC for 1-Year, 2-Year, and 3-Year OS predictions using the MT model in the validation cohort.

To further control for confounding factors, we conducted multivariate Cox regression analyses on variables selected through univariate Cox regression, LASSO regression, and RSF analysis. We employed a backward selection method, retaining variables with a p-value less than 0.05 in the final models. Consequently, we constructed three predictive models based on Cox regression, LASSO regression, and RSF, with results presented in **Table 3**. The bootstrap C-index for the training dataset was as follows: Cox regression model 0.705 (95% CI 0.676, 0.744), LASSO regression model 0.700 (95% CI 0.671, 0.741), and RSF model 0.691 (95% CI 0.661, 0.726). For the validation dataset, the bootstrap C-index was: Cox regression model 0.664 (95% CI 0.634, 0.725), LASSO regression model 0.668 (95% CI 0.626, 0.722), and RSF model 0.662 (95% CI 0.622, 0.712). Among these, the LASSO regression model demonstrated the highest bootstrap C-index on the validation dataset. Therefore, this study ultimately adopts the LASSO regression model for predicting survival outcomes in patients with advanced non-small cell lung cancer. The final model included the following variables: N stage, NLR, D-dimer, NSE, SCC, driver alterations and first-line treatment. In the training dataset, the model demonstrated relatively high predictive accuracy, with AUCs of 0.765 (95% CI 0.705, 0.824) for 1-year, 0.753 (95% CI 0.7, 0.806) for 2-year, and 0.806 (95% CI 0.755, 0.857) for 3-year survival predictions, indicating strong short to medium-term predictive capabilities, particularly for 3-year outcomes. In contrast, on the validation dataset, performance slightly declined but remained effective, with AUCs of 0.707 (95% CI 0.633, 0.781) for 1-year, 0.691 (95% CI 0.616, 0.765) for 2-year, and 0.696 (95% CI 0.611, 0.781) for 3-year predictions, affirming the model’s reasonable predictive power on an independent sample set, see **Figure 4**.

**Table 3:** Multivariate Cox regression analysis of variables selected by univariate Cox regression, LASSO regression, and random survival forest.

characteristics	Cox regression			LASSO regression			Random survival forest			Method
	HR	CI	P	HR	CI	P	HR	CI	P	
N (<N2 vs. ≥N2)	1.558	1.095-2.218	0.014	1.617	1.146-2.282	0.006	1.538	1.090-2.170	0.014	backward
Brain metastasis (No vs. Yes)	1.525	1.152-2.018	0.003							backward
NLR (<2.565 vs. ≥2.565)	1.607	1.194-2.162	0.002	1.671	1.251-2.234	0.001	1.569	1.174-2.097	0.002	backward
LDH (<230.5 vs. ≥230.5), (U/L)	1.233	0.929-1.635	0.147				1.298	0.978-1.725	0.071	backward
D-dimer (<300.0 vs. ≥300.0), (ng/ml)	1.496	1.110-2.015	0.008	1.506	1.140-1.990	0.004	1.42	1.067-1.89	0.016	backward
NSE (<5.605 vs. ≥5.605), (μg/L)	1.417	1.077-1.863	0.013	1.538	1.181-2.004	0.001	1.38	1.049-1.815	0.021	backward
SCC (<0.445 vs. ≥0.445), (ng/ml)	1.539	1.113-2.128	0.009	1.602	1.169-2.194	0.003				backward
Ki67										
(<50% vs. ≥50%)	1.4	1.002-1.956	0.048							backward
(<50% vs. Unknown)	1.085	0.779-1.510	0.630							backward
driver alterations										
(Yes vs. No)	2.02	1.232-3.312	0.005	2.190	1.337-3.586	0.002	1.872	1.377-2.544	0	backward
(Yes vs. Unknown)	3.071	1.824-5.171	0	3.259	1.941-5.472	0	3.051	2.162-4.304	0	backward
first line treatment										
(Chemotherapy vs. Targeted Therapy)	0.993	0.616-1.600	0.976	1.102	0.685-1.773	0.688				backward
(Chemotherapy vs. Immunotherapy)	0.541	0.350-0.835	0.006	0.585	0.38-0.902	0.015				backward

Furthermore, we utilized a validation dataset to evaluate the advanced NSCLC prediction model developed by Tao Wang and his team (referred to as the TW model) (7), and compared its performance with that of the LASSO regression model. The validation results indicated that the bootstrap C-index for the TW model was 0.619 (95%CI 0.581, 0.675), which was lower than that of the LASSO regression model. For a comparison of the ROC curves of the TW model and the LASSO regression model on the validation dataset, see **Figure 4**.

**Figure 5:**
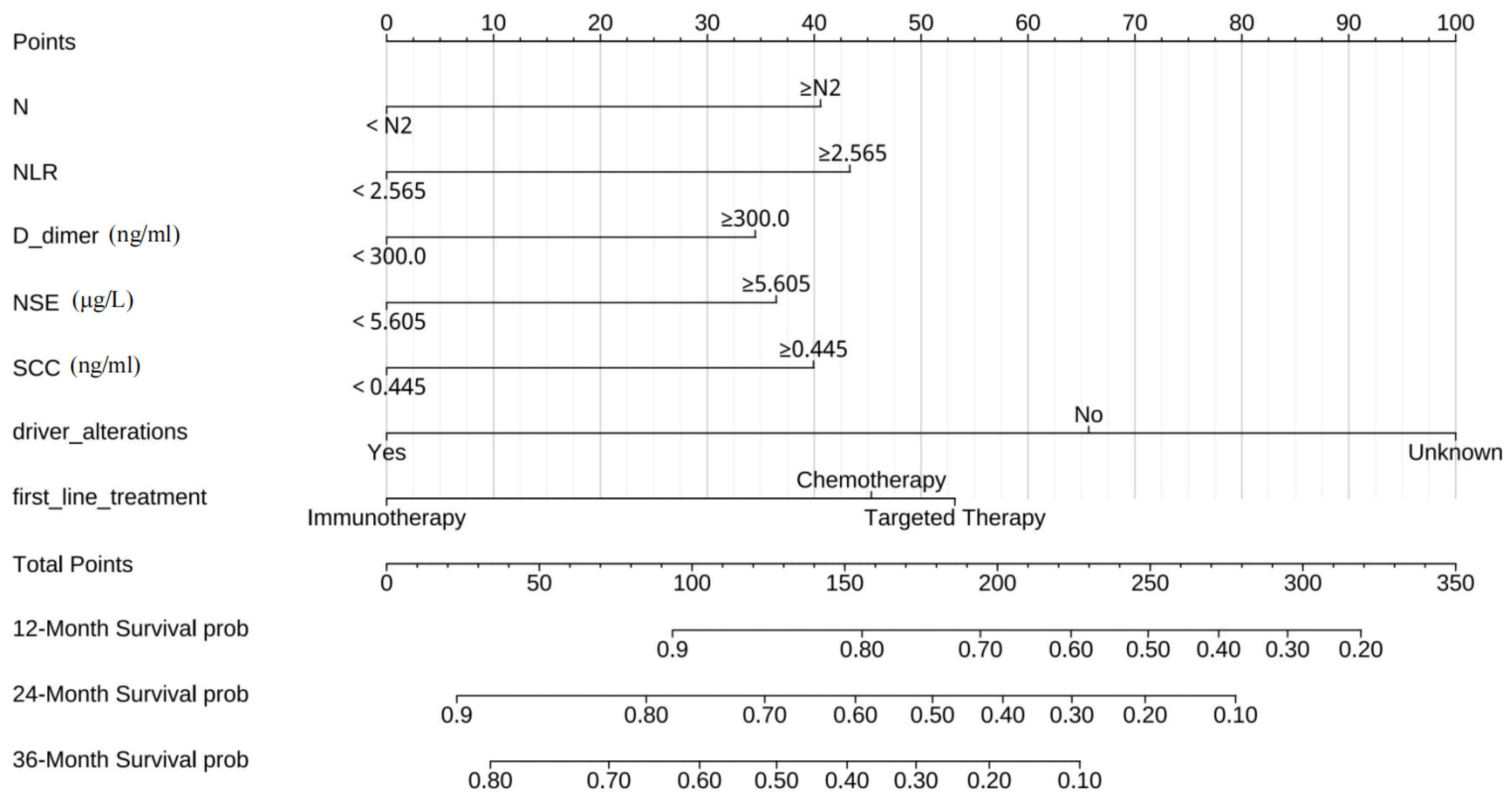
The nomogram to predict individual prognosis in advanced non-small cell lung cancer.

To further facilitate the application of our results, we created a nomogram based on the LASSO regression model. This graphical tool simplifies the estimation of individual survival probabilities for advanced non-small cell lung cancer patients, particularly focusing on their 1-3 year survival rates. It enables clinicians to make more informed decisions regarding prognosis and treatment strategies. The specific nomogram for these time points is illustrated in **Figure 5**.

### 3.3 Validation and clinical application of Lasso regression model

**Figure 6:**
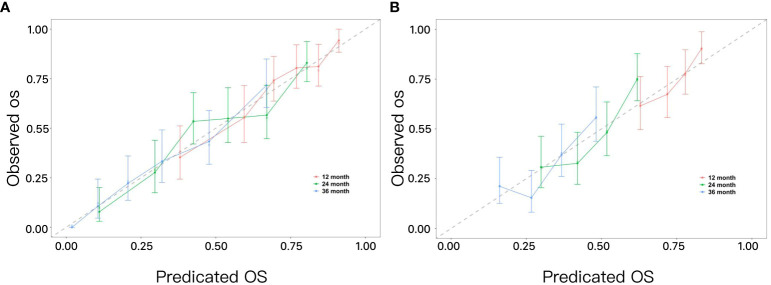
**(A)** Calibration curves for predicting 1-year, 2-year, and 3-year OS in advanced non-small cell lung cancer in the training set. **(B)** Calibration curves for predicting 1-year, 2-year, and 3-year OS in advanced non-small cell lung cancer in the validation set.

To evaluate the calibration of the nomogram, calibration plots were utilized, as shown in **Figure 6**. These plots visually demonstrate the model’s accuracy by depicting the correlation between the predicted probabilities and the actual observed outcomes across both the training and validation datasets. The analysis of these calibration curves indicates that the model’s estimations of 1-year, 2-year, and 3-year survival rates for patients with advanced NSCLC align closely with the observed survival rates.

**Figure 7:**
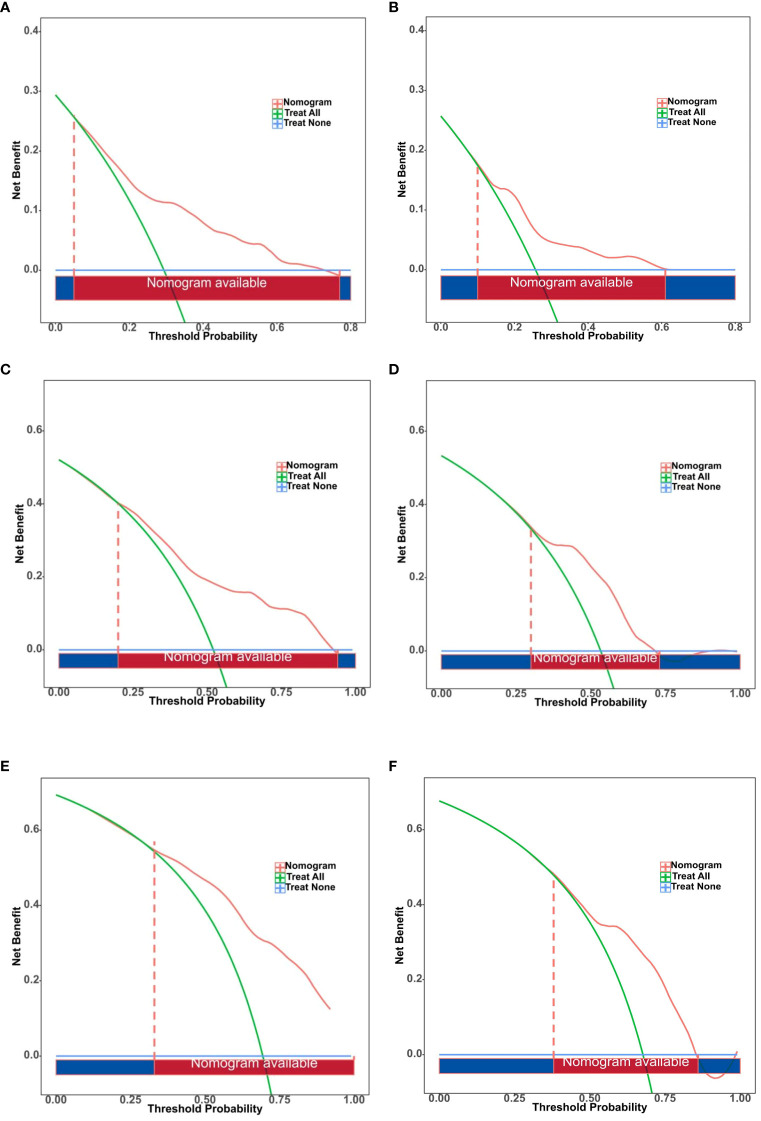
Decision curve analysis (DCA) of the nomogram for predicting individual prognosis in advanced non-small cell lung cancer. **(A)** DCA of nomogram for 1-Year survival rate predictions in the training set. **(B)** DCA of nomogram for 1-Year survival rate predictions in the validation set. **(C)** DCA of nomogram for 2-Year survival rate predictions in the training set. **(D)** DCA of nomogram for 2-Year survival rate predictions in the validation set. **(E)** DCA of nomogram for 3-Year survival rate predictions in the training Set. **(F)** DCA of nomogram for 3-Year survival rate predictions in the validation set.

The DCA of the nomogram for predicting individual prognosis in advanced NSCLC is detailed in **Figure 7**. For 1-year survival rates, the DCA threshold range was 5%—77% in the training dataset and 10%—61% in the validation dataset. For 2-year survival rates, the range was 20%—94% in the training dataset and 30%—73% in the validation dataset. For 3-year survival rates, the thresholds spanned from 33%—100% in the training dataset to 38%—86% in the validation dataset. These results underscore that the model provides clinically valuable information for decision-making at various prognostic time points.

**Figure 8:**
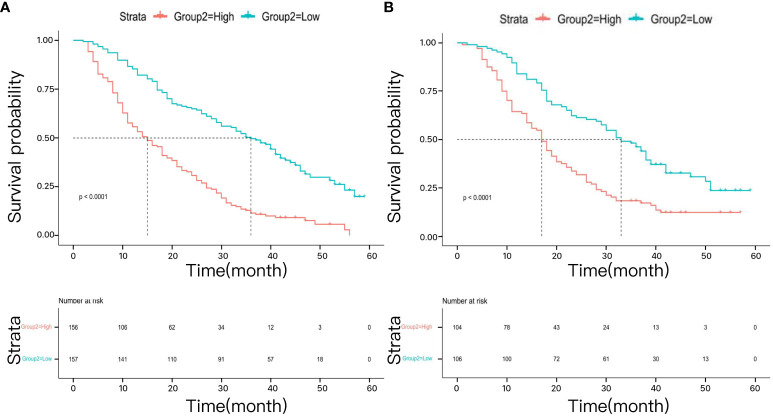
The survival analysis for different risk groups in the training cohort **(A)** and validation cohort **(B)**.

Utilizing the median risk score derived from the developed model, patients with advanced NSCLC were stratified into high-risk and low-risk groups. In the training dataset, the median OS for the high-risk group was 15 months (95% CI 12, 18), while it was 18 months (95% CI 15, 22) in the validation dataset. Conversely, the low-risk group demonstrated a median OS of 36 months (95% CI 30, 42) in the training dataset and 32 months (95% CI 24, 38) in the validation dataset. The Kaplan-Meier survival curves for both the training and validation datasets revealed that the median OS was significantly better in the low-risk group compared to the high-risk group, with statistically significant differences observed in both datasets (p < 0.0001, **Figure 8**).

## 4 Discussion

Over the past decade, significant advancements have been made in the treatment of advanced NSCLC, particularly in the areas of targeted therapy and immunotherapy. These developments have provided unprecedented survival benefits for some patients, occasionally extending life expectancy by several years (18–21). However, the overall prognosis for advanced NSCLC remains challenging. Currently, the AJCC Tumor-Node-Metastasis (TNM) staging system is the most commonly used prognostic model. Despite its prevalence, the anatomically based staging method fails to consider crucial factors such as genetic mutation types, histological subtypes, and treatment modalities, thereby exhibiting limitations in prognostic accuracy across diverse cancer types (22–24). This emphasizes the significance of developing novel, precise, and dependable prognostic models that incorporate a more comprehensive array of clinical and pathological characteristics to effectively anticipate disease outcomes and treatment responses, thereby facilitating personalized therapeutic strategies for patients.

Our study utilized three commonly employed methods in constructing survival prognosis models: Cox regression, LASSO regression, and RSF. Cox regression, a conventional statistical approach, is well-suited for analyzing survival data and considering the impact of multiple covariates. However, it encounters challenges in dealing with multicollinearity among variables (25). In contrast, machine learning techniques such as LASSO regression and RSF exhibit enhanced adaptability in handling intricate data. LASSO regression addresses issues of multicollinearity and overfitting by incorporating a regularization term that facilitates feature selection (26). RSF, an ensemble learning method, effectively manages high-dimensional data and nonlinear relationships, thereby improving model robustness and accuracy through the integration of multiple decision trees (27).

When comparing the performance of these three models on the validation dataset, we observed that LASSO regression achieved a Bootstrap C-index of 0.668, slightly surpassing the Cox model (0.664) and the RSF model (0.662). This marginal difference suggests a slight advantage of LASSO regression in handling unseen data. Moreover, LASSO regression’s variable selection capability simplifies and enhances interpretability and generalizability, which is particularly crucial when dealing with complex datasets containing multiple predictors. Therefore, despite similar overall performance among the three models, LASSO regression is considered an optimal choice due to its slight performance edge on the validation dataset and superior variable selection capabilities.

The LASSO regression model demonstrated substantial clinical utility in both the training and validation datasets within the DCA (**Figure 7**). Notably, it exhibited significant net benefits in predicting 1-year survival rates, effectively supporting short-term clinical decision-making. Although the range of decision thresholds for predicting 2-year and 3-year survival rates was relatively narrow in the validation dataset, our results still indicate that this model holds potential for practical application in medium to long-term prognostic predictions. Furthermore, we observed significant differences in median OS times between risk groups stratified by the model (p < 0.0001), further validating its efficacy in distinguishing patients with varying risk levels (**Figure 8**).

Nomograms are decision-support tool that visually represents data, renowned for their practicality and intuitive nature in clinical medicine. These graphical tools simplify complex clinical data and statistical models into easily comprehensible visual formats, enabling physicians to swiftly grasp a patient’s health status and prognosis. For instance, in our model, an advanced NSCLC patient has an N stage of N3 (40.7 points), an NLR exceeding 2.565 (43.5 points), a D-dimer level below 300.0 ng/ml (0 points), an NSE level surpassing 5.605 μg/L (37 points), an SCC level above 0.445 ng/ml (40 points), the presence of driver alterations (0 points), and is receiving targeted therapy as the first-line treatment (52.8 points). The cumulative score of 214 corresponds to a one-year survival rate of 63.3%, a two-year survival rate of 34.4%, and a three-year survival rate of 14.2%.

In our study, we employed three distinct variable selection methods to identify clinical features that significantly impact the prognosis of NSCLC. Notably, five variables—N stage, NLR, D-dimer, NSE, and driver alterations—were consistently selected across all three methods. This finding highlights the potential importance of these variables in the prognostic assessment of NSCLC.

As part of cancer staging, N stage directly reflects the extent and severity of cancer spread, and its consistent selection across all methods validates its stability and reliability as a prognostic marker. The NLR refers to the ratio of neutrophils to lymphocytes and serves as an indicator of systemic inflammatory response. It is associated with survival rates across various types of cancer (28–30). Our study further confirms the effectiveness of NLR as an independent prognostic factor, wherein elevated NLR levels may signify immune suppression and inflammation that foster tumor progression. D-dimer, a marker of coagulation and fibrinolytic system activity, often correlates with the progression of malignancies (31). Tumor cells can activate the coagulation system through various mechanisms, such as releasing procoagulant factors, leading to elevated D-dimer levels. This activation can further promote tumor cell proliferation and metastasis (32, 33), thus establishing a detrimental cycle. Therefore, D-dimer levels can serve as a useful biomarker in tumor management, helping physicians assess disease severity, prognosis, and treatment efficacy. However, since elevated D-dimer can also result from non-neoplastic conditions, its use as a cancer marker must be approached with caution, typically in conjunction with other clinical information and findings. NSE is an enzyme expressed in neural tissues and neuroendocrine cells and is a commonly used tumor marker. Although more commonly associated with small cell lung cancer (SCLC), NSE also has applications in the diagnosis and prognosis of NSCLC. Patients with high NSE levels generally exhibit higher risks of recurrence or metastasis, indicating a poorer prognosis (34, 35). Ultimately, the identification of driver alterations is crucial not only for understanding the biological behavior of tumors but also for determining patient responses to specific treatment regimens. Our study indicates that patients lacking driver gene mutations have a 2.19-fold higher risk of mortality compared to those with driver gene mutations. This finding underscores the significant impact of targeted therapies in extending the survival of patients with genetic mutations. It supports the role of personalized medicine and highlights the application value of molecular profiling in cancer treatment.

Furthermore, the model incorporates SCC and first-line treatment protocols to enhance the accuracy of prognosis assessment in patients with Non-Small Cell Lung Cancer (NSCLC). SCC antigen is a commonly used tumor marker in patients with squamous cell carcinoma, and elevated levels are closely associated with tumor burden, disease progression, and poor prognosis (36, 37). Particularly in NSCLC, especially within the squamous cell carcinoma subtype, measuring SCC can provide critical information about tumor behavior and treatment response (38–40).

In clinical practice, the selection of first-line treatment for NSCLC is based on multiple factors, including but not limited to the genetic expression type of the tumor, the overall health status of the patient, and treatment preferences. Typically, targeted therapy, immunotherapy, or chemotherapy are employed as first-line treatments for most NSCLC patients based on the molecular characteristics of the tumor and individual circumstances. For instance, NSCLC patients with EGFR mutations or ALK fusions may derive benefits from tailored therapies targeting these alterations. However, some patients whose genetic expression types have not been clearly identified may still opt for targeted drugs as their preferred first-line treatment, though they may not achieve the anticipated therapeutic benefits. This situation might explain why our analysis shows no significant statistical difference between selecting targeted drugs and chemotherapy as first-line treatments. This highlights the significance of genetic testing and personalized treatment approaches employed by physicians. Furthermore, our analysis demonstrates that immunotherapy presents a 41.5% reduction in mortality risk compared to chemotherapy, further confirming the potential of immunotherapy in the treatment of NSCLC. With advancements in scientific knowledge and an enhanced comprehension of the tumor microenvironment, immunotherapy has progressively emerged as a pivotal element within treatment strategies for various cancer types. Particularly within NSCLC, the application of immunotherapy offers new hope for patients.

Despite employing various statistical and machine learning techniques to enhance the accuracy of our prognostic models, there are several limitations that need to be addressed. Firstly, our study relies on a dataset from a single center, which may restrict the generalizability and extrapolation of our models. Patients from diverse regions and populations may exhibit significant variations in genetic backgrounds, lifestyles, and treatment adherence, all of which could impact the predictive power of our models. Secondly, although we made efforts to collect a comprehensive set of clinical variables, certain critical biomarkers or patient characteristics such as quality of life, mental health status, and socioeconomic factors might have been overlooked. These elements represent potential key factors influencing the prognosis of lung cancer patients but are often unavailable in retrospective cases. Thirdly, although we analyzed the comorbidities of the patients, the retrospective nature of our study limited our ability to conduct in-depth analyses of each specific comorbidity. This limitation may have prevented us from fully capturing the unique impact of each comorbidity on prognosis. Fourth, our model has not undergone external validation, which is a significant limitation of this study. Although our internal validation results demonstrate that the model performs well in predicting the prognosis of NSCLC patients, the lack of external validation limits the generalizability and reliability of the results. External validation is a crucial step in assessing the consistency of the model’s performance across different datasets and clinical settings. Fifth, the categorization of treatment regimens used in this study may be overly simplified. In actual clinical settings, treatment plans for patients are typically more complex and personalized, involving combinations of multiple drugs and dynamic adjustments in treatment strategies. This complexity is likely not adequately captured by the current model. Lastly, treatment selection bias is another limitation that must be acknowledged. In observational studies, treatment allocation is often non-random and may be influenced by patient baseline characteristics. Although we attempted to control for these factors using multivariable regression analysis, the potential for residual confounding remains. This bias could affect the model’s predictions and limit its applicability in different clinical scenarios. Therefore, future research should consider conducting prospective studies and using datasets from multiple centers and countries to enhance the universality and robustness of the models. Further exploration into additional potential influencing factors, including more biomarkers and quality of life indicators, as well as developing more comprehensive and dynamic tools for assessing treatment responses should be pursued to improve both comprehensiveness and practicality. Additionally, we plan to include more real-world data from patients with advanced NSCLC to perform subgroup analyses of different treatment regimens. By constructing prognostic models specific to each treatment group, we hope to further refine and improve our research results.

## 5 Conclusion

In conclusion, we have developed a robust predictive nomogram model specifically tailored to the unique characteristics of advanced NSCLC, enabling accurate prediction of individual survival probabilities with high levels of discrimination and agreement. To enhance the validity and applicability of our model, it is recommended to conduct large-scale and multicenter studies for further evaluation and validation.

## Data availability statement

The original contributions presented in the study are included in the article/supplementary material. Further inquiries can be directed to the corresponding authors.

## Ethics statement

The studies involving humans were approved by Shanxi Province Cancer Hospital Ethical Review Board. The studies were conducted in accordance with the local legislation and institutional requirements. Written informed consent for participation was not required from the participants or the participants’ legal guardians/next of kin in accordance with the national legislation and institutional requirements.

## Author contributions

YG: Conceptualization, Data curation, Formal analysis, Investigation, Writing – original draft, Writing – review & editing. LL: Data curation, Investigation, Writing – original draft. KZ: Conceptualization, Investigation, Writing – original draft. JD: Investigation, Writing – review & editing. JN: Investigation, Writing – review & editing. ZW: Funding acquisition, Methodology, Writing – review & editing. ZH: Methodology, Project administration, Supervision, Writing – review & editing.
